# The Multifeature Gait Score: An accurate way to assess gait quality

**DOI:** 10.1371/journal.pone.0185741

**Published:** 2017-10-19

**Authors:** Khaireddine Ben Mansour, Philippe Gorce, Nasser Rezzoug

**Affiliations:** Handibio—EA4322—Université de Toulon, Toulon–Var, La Garde cedex, France; Universite de Nantes, FRANCE

## Abstract

**Purpose:**

This study introduces a novel way to accurately assess gait quality. This new method called Multifeature Gait Score (MGS) is based on the computation of multiple parameters characterizing six aspects of gait (temporal, amplitude, variability, regularity, symmetry and complexity) quantified with one inertial sensor. According to the aspects described, parameters were aggregated into partial scores to indicate the altered aspect in the case of abnormal patterns. In order to evaluate the overall gait quality, partial scores were averaged to a global score.

**Methods:**

The MGS was computed for 3 groups namely: healthy adult (10 subjects), sedentary elderly (11 subjects) and active elderly (20 subjects). Data were gathered from an inertial sensor located at the lumbar region during two sessions of 12m walking.

**Results:**

The results based on ANOVA and Tukey tests showed that the partial scores with the exception of those which describe the symmetry aspect were able to discriminate between groups (p<0.05). This significant difference was also confirmed by the global score which shows a significantly lower value for the sedentary elderly group (3.58 ±1.15) compared to the healthy adults (5.19 ±0.84) and active elderly (4.82 ±1.26). In addition, the intersession repeatability of the elaborated global score was excellent (ICC = 0.93, % SEM = 10.81).

**Conclusion:**

The results obtained support the reliability and the relevance of the MGS as a novel method to characterize gait quality.

## Introduction

Clinical assessment tests such as Tinetti and Get Up and Go require the patient to perform several tasks and lead directly to a subjective outcome evaluating inter alia the gait quality which is considered as the deviation of gait from normative data. However, using instrumented tests involves a wide range of quantitative measures which may be of interest to many fields such as sports, reeducation and diagnosis of health status [1,2]. In a medical framework, these measures are used to monitor the progression of healing of patients or to test the effectiveness of a rehabilitation program [3]. They are also used in health diagnosis to identify certain pathologies, to assess the general health status [4,5] or to determine the effect of physical activity [6,7]. However, in clinical settings, the large number of parameters makes the analysis and interpretation complex. In order to simplify the daily practices of clinicians some studies have established methods to aggregate multiple parameters into a single value called "score" or "index" [8–12]. In the case of gait assessment, this numeric representation characterizes in a simpler way the degree of alteration of the walking pattern, assess the result of surgical intervention and evaluate the effect of a reeducation or physical activity program prescribed to improve the gait quality [13–15].

Previous studies [8–13] established scores or indexes based on the quantification of a set of biomechanical parameters characterizing only one aspect of the gait. Among these reliable scores, the Gait Variability Index (GVI), defined as an objective quantification of dynamic instability and deviation from asymptomatic gait pattern, rests on the quantification of the variability of nine spatiotemporal parameters [11]. The Functional Ambulation Performance Score (FAPS), considered as a quantitative measure, provides an alternative description of the degree of any alteration by computing five spatiotemporal parameters [11,16]. The Gait Deviation Index (GDI) and the Gait Deviation Index Kinetic (GDI-Kinetic) are estimated based only on the successive values of nine joint angles and nine kinetic parameters gathered from the lower limbs during a gait cycle, respectively [9,10]. These index have been developed to evaluate a specific aspect of gait. In clinical practice, several aspects are assessed conjointly to get a complete picture of the patient’s gait. Indeed, some people may conserve a gait pattern qualified as suitable when spatiotemporal parameters are taken into account while the movement of the joint angles are atypical [17]. Furthermore, the simultaneous computation of the set of the existent scores to establish a complete picture of the patient's gait requires different measurement systems such as force plates or optoelectronic systems available mostly in research settings.

Contrary to the scores described previously, the normalcy index, also called the Gillette Gait Index (GGI), incorporates sixteen parameters characterizing two different aspects of gait namely spatiotemporal (3 parameters) and kinematics (13 joint angles of the lower limbs) in order to quantify the difference between the gait of any individual and the reference group formed with healthy people [8]. The fact of considering more than one aspect of gait could be more representative of the gait quality. However, given that the overall capacity of an individual to walk is deduced from only the one value it will be impossible to define the altered aspect in the case of pathological gait. Thereby, it proves to be crucial to set up a novel method that qualifies each aspect separately in addition to the overall gait quality estimated through the global score. Nonetheless, based on the tools used to assess gait pattern, several aspects could be characterized through different parameters.

Habitually, gait assessment was made in a restricted environment (laboratory, clinical environment) even though results obtained in an ecological environment are more representative [18] and also people would be able to assess themselves continuously. In recent decades, the miniaturization and the extension of Micro-Electro-Mechanical Systems (MEMS) technology has led to lightweight, portable and especially low cost inertial sensors which are a reliable alternative to compute biomechanical parameters related to several aspects of gait even in the outdoors [19,20]. Such technology allows the quantification of the several gait aspects covered by the relevant existing scores but with a unique device instead of several laboratory equipment.

Within this framework, this article presents a new method based on one inertial sensor to accurately assess the alteration of the gait pattern or its improvement following a specific treatment based on different aspects of gait computed with only one inertial sensor. This method is based on the computation of a global score relating the overall gait quality and partial scores to indicate the altered aspect in the case of a pathological pattern.

## Methods

### 1. Participants

Forty-one participants forming three groups were included in the current study. The first was made of 10 healthy adults (4 men and 6 women, Mean ±SD: 27 ±3 years, 1.74 ±0.07 m and 68 ±13 kg), the second with 11 sedentary elderly which reported none physical activity (4 men and 7 women, 66 ±5 years, 1.61 ±0.06 m and 73 ±12 kg) and the third with 20 active elderly who practiced, within a sports association, Nordic walking regularly two times a week and during minimum one hour per session (4 men and 16 women, 62 ±4 years, 1.65 ±0.05 m and 67 ±14 kg). None of the participants used any technical assistance or reported difficulty in walking. The experiment was approved by the local ethics committee of the University of Toulon and conducted according to the principles expressed in the Declaration of Helsinki. Each volunteer signed a written informed consent.

### 2. Procedure

The participants stood behind a line drawn on the floor, arms beside the body. After a verbal instruction, they were asked to walk at self-selected speed and to stop after having crossed a second line drawn on the floor 12 m from the first line. According to previous studies, this distance was sufficient to obtain a steady state walking [21]. Then, participants had to come back to the starting position for the next trial. In order to have a more representative pattern and a representative estimated variability, each subject performed three trials during two different sessions scheduled at the same time of day and separated by one week. For each trial the two first and last steps were excluded.

### 3. Instrumentation

One inertial sensor was mounted with a belt and double sided tape on the L3-L4 inter-vertebral level. The purpose was to estimate the gait quality based on the pattern of acceleration (200 Hz; ±4 g; resolution: 7.8 mg) and angular velocity (200 Hz; ±250 deg.s^-1^; sensitivity accuracy: ±2%) gathered from a 3D capacitive accelerometer (MMA8453Q, Free scale Semiconductor, Austin, Texas, USA) and 3D gyroscope (L3G4200D, STMicroelectronics, Geneva, Switzerland), respectively. Accelerometric data were low pass filtered (zero lag 4^th^ order Butterworth filter, cut-off frequency (fc) = 30 Hz) [22] while angular velocity data were high pass (fc = 0.25 Hz) and low pass (fc = 30 Hz) filtered (zero lag 1^st^ order Butterworth filter) [23].

### 4. Process of the computation of the Multifeature Gait Score

**Fig 1:**
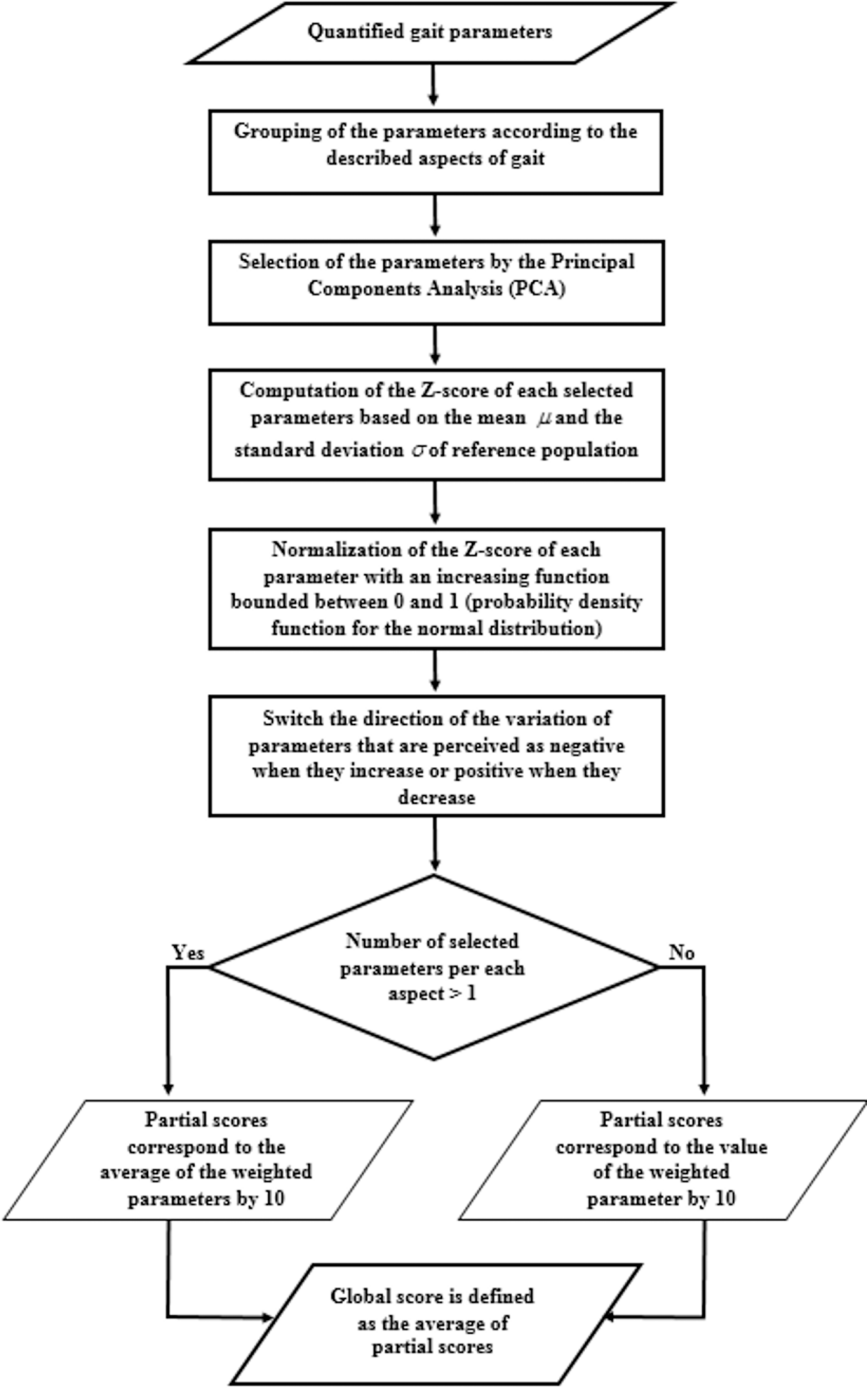
Flowchart for the quantification of the Multifeature Gait Score.

The formalization of this new method called Multifeature Gait Score (MGS) was carried out in several steps (Fig 1).

#### 4.1 Computed biomechanical parameters

In order to be more robust and more representative of the gait quality the MGS, contrary to established methods, includes parameters characterizing multiple aspects of gait. In fact, based on angular velocity and accelerometric signals gathered at the lumbar region, six different aspects of gait were assessed:

Amplitude: the amplitude of the inertial signals were quantified from three dimensions (anteroposterior «AP», vertical «V», mediolateral «ML») and the Euclidean Norm «N» through the measurement of the range (mean of the ranges computed per each step) and the Root Mean Square «RMS» [24]. The choice of this aspect is made in view of its sensitivity to the gait alteration during senescence [25].

Temporal: following the detection of the gait events from the AP and V components of the lumbar acceleration, the subsequent temporal parameters were quantified: stance, swing, double support, step and stride duration [22]. These parameters are known to fluctuate according to physical condition [26,27] and age [20,28].

Distribution: this aspect was grounded in the quantification of the skewness (asymmetry of the amplitude distribution) and the kurtosis (the distribution of amplitudes around the mean amplitude) from the three dimensions and N [29]. This aspect was also considered because it can convey information about the walking pattern. In fact, skewness and Kurtosis are able to distinguish between healthy and pathological groups [29].

Complexity: the complexity of the temporal series of the three components and N of the angular velocity and accelerometric signals was estimated by using the sample entropy (SamEn) [30]. It has been found that this measure of the complexity and predictability of temporal series decreases with age or pathology [29,30].

Symmetry: The symmetry represents the similarity between the average of the same parameter quantified for the left and right limb [31]. Gait symmetry is a good indicator of the gait quality and is considered to depend on physical and neurological functions [32]. In the current study, symmetry was estimated based on the temporal parameters.

Regularity: The regularity is a measurement of the similarity of the parameter quantified from the same side for two successive steps [31]. As symmetry, regularity decline in the case of presence of physical or neurological dysfunction [32]. Regularity was also estimated from temporal parameters.

#### 4.2 Principal component analysis

Parameters characterizing the same aspect of gait may be redundant. For this reason, a multivariate method called the Principal Component Analysis (PCA), was used to reduce the number of parameters by keeping only the independent ones. Given the differences in the magnitudes of scales and the measuring units of the computed parameters, the PCA was performed from the correlation matrix [33,34]. This matrix is none other than the covariance matrix of standardized variables [35]. The PCA was applied to a two-dimensional table (**i** x **j**) associating **i** individuals and **j** parameters. The 21 individuals in the current study were distributed in rows and the parameters in columns. To determine the number of the principal components (PC) considered, the method proposed by Kaiser (1960) [36] and Joliffe (1972) [37] was applied. This method proposes the consideration of only the PCs whose eigenvalues are greater than or equal to 1. Thereafter, the correlation coefficients (R) between the selected PC and each parameter are computed. Initially, only variables that have an absolute value of R greater than or equal to 0.4 (|R| ≥ 0.4) with p the probability that the variables and the PC are correlated being less than 5% (p<0.05) are considered. Thereafter, per each aspect and each PC, only the parameter presenting the highest value of R was retained.

#### 4.3 Elaboration of the partial scores

Once the redundant parameters had been simplified, the calculation of the partial scores was performed in three steps. The first consisted in computing the Z-score of each parameter based on the mean *μ* and the standard deviation *σ* of the reference population approximated from the estimation of the sample mean ($\bar{X}$) and standard deviation (*s*) of the healthy adult group (Eq 1). After that, an increasing function *f* (the cumulative probability density function of the normal distribution) set between zero and one was applied to standardize the parameters. Finally, the different normalized parameters associated with the same aspect of walking were averaged and weighted by a coefficient to obtain a partial score between zero and ten (Eq 2).

$$z_{i}=\frac{X_{i}-\mu_{i}}{\sigma_{i}}$$ (1)

$$S_{Partial}=\frac{10}{P}\times \sum_{i=1}^{P}f\left(z_{i}\right)$$ (2)

*P* represents the number of the kept parameters per each aspects.

Based on this method, a higher partial score is synonymous of a better gait quality. However, by definition, the gait biomechanical parameters could decrease or increase in healthy people. By considering that the desired values are those of a healthy adult and that an increase in the score is perceived as positive, it is necessary, before averaging the parameters representative of the same aspect, to switch the direction of variation of parameters that are perceived as negative when they increase or positive when they decrease. The parameters concerned were modified as follows (Eq 3):

$$Y_{i}=1-X_{i}$$ (3)

**Fig 2:**
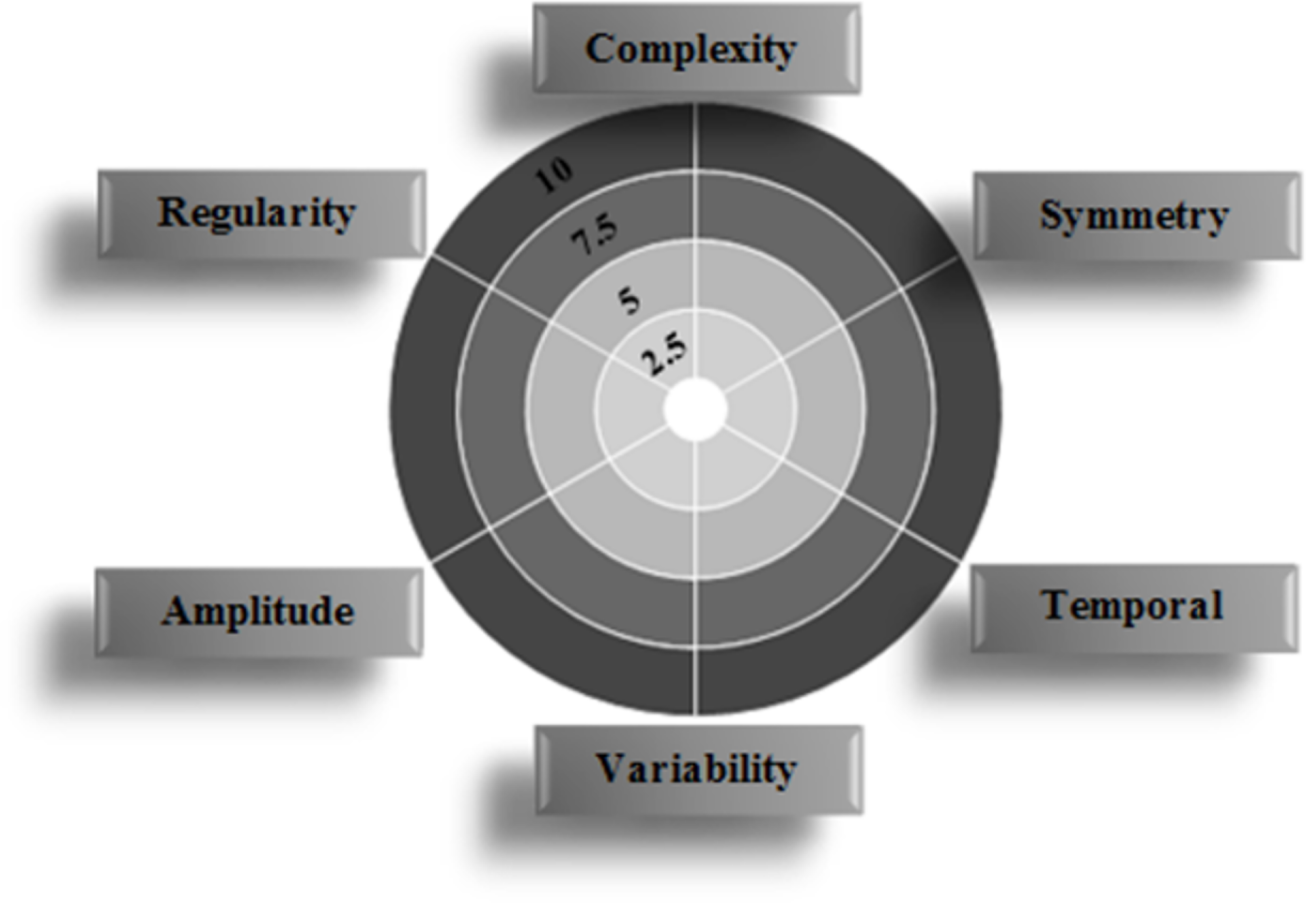
Example of polar diagram grounded on six aspects of gait.

To facilitate the reading of partial scores, a polar representation was established. Each radius characterizes one partial score (Fig 2).

#### 4.4 Elaboration of the global scores

A global score representative of the overall quality of gait was calculated by considering the average of the estimated partial scores (Eq 4):

$$S_{Global}=\frac{1}{N}\sum_{i=1}^{N}S_{partial}{}_{{}_{i}}$$ (4)

N represents the number of the computed aspects.

### 5. Data analysis

Once the partial and global scores had been computed (data in S1 Table), a one-way ANOVA considering the group (GHA, GNW, GSE) as the independent variable was conducted, followed by post-hoc Tukey tests if necessary. Subsequently, to assess the intersession repeatability of the MGS, the intraclass correlation coefficients (model 2,K) and the standard error of measurement expressed as a percentage of the mean value of the score were quantified (% SEM). Based on previous studies [38–40], the level of repeatability will be qualified as:

- Excellent if: ICC>0.75
- Fair to good if: 0.4<ICC<0.75
- Low if: ICC<0.4

## Results

### 1. Reducing of the parameters through PCA

Following the application of the PCA, eight PC whose eigenvalues were greater than or equal to one were considered. These PC explain 84% of the total variance of the quantified parameters. Based on the two first steps of the development process of the MGS (Fig 1), only ten parameters were selected to characterize the six aspects described (Table 1).

**Table 1:** Illustration for each aspect of the independent parameters.

Aspects	Parameters	Correlation coefficient
Temporal	Duration of the stance phase	CP1: 0.82
	Duration of the double support phase	CP3: 0.82
Symmetry	Symmetry of the swing phase	CP2: 0.89
	Symmetry of the double support phase	CP5: 0.72
	Symmetry of the stride	CP7: 0.48
Regularity	Regularity of the Stride	CP6: 0.65
Complexity	Sample Entropy of the ML component of the angular velocity	CP4: 0.65
Amplitude	Rms of the N of the acceleration	CP1: 0.94
Distribution	Skewness of the Vertical component of acceleration	CP3: 0.62
	Skewness of the norm of acceleration	CP4: 0.59

### 2. Elaborated partial scores

ANOVA showed that the majority of the quantified aspects were able to discriminate between groups. In fact, contrary to the partial scores computed from parameters describing the symmetry aspect, the other partial scores showed significant differences between the groups considered (p<0.05).

**Fig 3:**
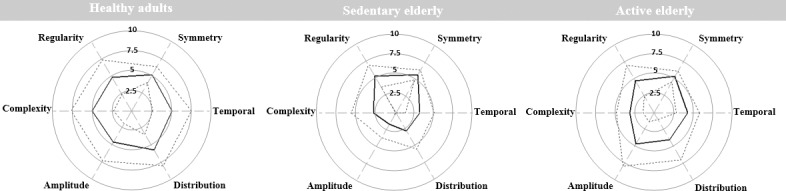
Polar representation of the partial scores quantified for each group. The black line corresponds to the mean of partial scores. Dashed line corresponds to ± SD.

Tukey tests showed that the partial scores computed from parameters characterizing the temporal, regularity and amplitude aspects were significantly lower in the sedentary elderly compared to the active elderly and healthy adult groups (p<0.05). Concerning the complexity aspect, Tukey tests showed that the related partial scores were significantly lower in the sedentary and active elderly compared to the healthy adults (p<0.05). Furthermore, Tukey tests showed that the partial scores computed for the distribution aspect differentiated only healthy adults from the sedentary elderly. This group presents the lowest value (p<0.05). The polar representation of the partial scores quantified for each group is presented in Fig 3.

### 3. Elaborated global scores

ANOVA showed a significant difference between groups through the quantified global score (F(2, 41) = 7.442, p = 0.001).

Tukey tests showed that the global score quantified for the sedentary elderly group (3.58 ±1.15) was significantly lower (p<0.05) than that of the healthy adults (5.19 ±0.84) and active elderly (4.82 ±1.26).

### 4. Assessment of the repeatability of the MGS

#### 4.1 Repeatability of the global score

The assessment of the ICC_2,K_ (with k = 1) and the SEM revealed that the intersession repeatability of the elaborated global score was excellent (ICC = 0.93, % SEM = 10.81).

#### 4.2 Repeatability of the partial scores

The assessment of the ICC_2,K_ (with k = 1) and the SEM revealed that the intersession repeatability of the elaborated partial scores was excellent for temporal (ICC = 0.91, % SEM = 19.44), complexity (ICC = 0.87, % SEM = 35.46), amplitude (ICC = 0.97, % SEM = 17.99) and distribution aspects (ICC = 0.91, % SEM = 31.35). Concerning the elaborated partial scores characterizing the symmetry (ICC = 0.64, % SEM = 13.83) and regularity aspects (ICC = 0.70, % SEM = 32.40) the intersession repeatability was fair to good.

## Discussion

The MGS was developed to allow an objective estimation of gait quality. Contrary to existing scoring methods, the MGS takes into account several aspects of gait computed with only one wearable system and one assessment for the subject. In fact, the estimation of the gait quality is more accurate with a higher number of computed aspects. To estimate the overall gait quality and to synthetize the parameters describing the same aspect of gait, global and partial scores were quantified respectively. The interest of quantifying partial scores in the case of a pathological gait is to define the altered aspect and to allow tracking of its improvement following a specific treatment or a regular physical activity.

In this study, the aspects described were based on biomechanical parameters computed through signals gathered from an inertial sensor located at the lumbar region. These parameters were deemed sensitive to the alteration and the amelioration of the gait quality [20,29,41]. Through the quantification of the partial and global scores, the MGS showed potential to objectively represent the gait quality as a whole or by aspects. For each aspect, the correlation between interrelated biomechanical parameters was taken into account. In fact, after proceeding with a multivariate analysis (PCA) only interdependent parameters were synthetized into partial scores to indicate how much the given aspect differs from the reference group. The sum of the partial scores, namely the global score, is more general and based on a single value it indicates the amount of the deviation of the gait pattern from a healthy adult. The results of the current study show that unlike the other scores, partial scores describing the symmetry aspect do not discriminate groups. This is due to the fact that none of the participants had suffered from pathologies inducing gait asymmetry such as a stroke [42]. Results also showed that the partial scores describing the temporal, regularity and amplitude aspects differ significantly between groups. In fact, for these three aspects, the sedentary elderly present lower scores compared to the active elderly and healthy adult groups. These findings corroborate those reported in previous research which showed that aging and regular physical activity have a deleterious and beneficial effect respectively on the parameters considered following the PCA [27,42,43]. Concerning complexity and distribution aspects, the related partial scores differentiate only the reference group from the two others. For complexity, The low value in elderly groups compared to the healthy adult group reflects the restriction of the diversity of movement strategies [43]. The absence of significant differences between the sedentary and active elderly for complexity and distribution aspects may be due to the fact that the predictability of temporal series and the statistical distribution are not sensitive to the effect of a regular physical activity contrary to the modifications occurring during senescence.

Concerning the global score, results showed that the MGS is a relevant tool to estimate the overall gait quality. In fact, the global score quantified for the sedentary elderly was significantly lower than that of the healthy adult. This outcome highlights the deterioration of the gait pattern during aging. Moreover, the global score showed a significantly higher value for healthy adults and the active elderly compared to the sedentary elderly group marking the sensitivity to the improvements of gait quality following a regular practice of physical activity.

In order to have an accurate assessment of the gait quality, the intersession repeatability was computed to ensure that the quantified scores really characterize the walking pattern. The findings showed that the repeatability of this novel method was excellent (ICC = 0.93 and % SEM = 10.81) which confirms the suitability of the MGS for gait assessment or monitoring. In addition, based on the fact that the partial scores are presented in the form of a polar diagram, clinicians could easily situate the partial scores of the assessed subject compared to the reference group. In the case of monitoring, given that the health state of the individual is known it would be more appropriate to preselect the parameters according to their sensitivity to the pathology. This preselection may be made based on previously established parameters or after a parameter sensitivity study. In fact, the relevance of the scores and their capacity to discriminate between a reference pattern and an altered one depends heavily on the quantified parameters. We assume that contrary to the evaluated parameters, the elaborated method is not specific to any cohort. In fact, with suitable parameters, the methodology of the MGS should be relevant to characterize gait quality independently from the age and the health state. However the potential limitation of this study is the small number of healthy adults which may impact the accuracy of the MGS. A further limitation may be the absence of a group with persons suffering from pathologies that affect symmetry. For clinicians, the need of statistical analysis to discriminate a healthy from a pathological pattern also constitutes a restriction as patients are assessed individually.

In fact, this study corresponds to a first step and future studies should continue to broaden the field of this novel method and maximize the number of healthy adults to minimize the standard deviation of the reference pattern. Moreover, asymmetric patients should be included and a threshold for each aspect from which healthy and pathological patterns can be discriminated should be established.

## Conclusion

To our knowledge, this is the first study assessing multiple aspects of gait based on one wearable sensor which can lead to gait assessment in outdoor conditions. The results obtained in this current study demonstrate that the MGS is accurate in characterizing gait quality. The MGS reports the deterioration during senescence and the amelioration through a regular physical activity.

## Supporting information

S1 Table Illustration of the partial and global scores computed for each participant. (PDF)
