# Supplementary material for: The Multifeature Gait Score: An accurate way to assess gait quality
Source: PLoS One. 2017 Oct 19;12(10):e0185741. doi: 10.1371/journal.pone.0185741 (PMC5648116; doi:10.1371/journal.pone.0185741)
Supplement: S1 Table — (PDF) [file pone.0185741.s001.pdf]

| Group | Subject | Partiel scores: Temporal | Symmetry | Regularity | Complexity | Amplitude | Distribution | Global scores |
|---|---|---|---|---|---|---|---|---|
| Group of Healthy Adults (GHA) | 1 | 7,28 | 5,93 | 0,79 | 3,92 | 9,30 | 6,77 | 5,67 |
| | 2 | 8,14 | 3,07 | 5,48 | 6,86 | 7,96 | 0,47 | 5,33 |
| | 3 | 4,17 | 6,09 | 7,24 | 5,37 | 1,60 | 5,30 | 4,96 |
| | 4 | 3,02 | 6,37 | 6,60 | 7,69 | 5,18 | 5,18 | 5,67 |
| | 5 | 2,50 | 4,04 | 6,68 | 7,44 | 3,36 | 8,99 | 5,50 |
| | 6 | 5,44 | 6,11 | 3,02 | 4,49 | 2,62 | 6,56 | 4,71 |
| | 7 | 1,91 | 6,76 | 5,31 | 7,41 | 1,47 | 4,20 | 4,51 |
| | 8 | 9,52 | 5,72 | 1,41 | 0,64 | 4,37 | 7,10 | 4,79 |
| | 9 | 4,75 | 4,14 | 8,66 | 6,31 | 4,90 | 7,26 | 6,00 |
| | 10 | 5,51 | 5,13 | 4,58 | 0,64 | 6,60 | 6,07 | 4,76 |
| Group of Sedentary Elderly (GHA) | 1 | 0,42 | 5,89 | 6,18 | 1,03 | 0,34 | 0,03 | 2,32 |
| | 2 | 2,58 | 4,92 | 4,18 | 1,98 | 0,50 | 2,11 | 2,71 |
| | 3 | 5,96 | 6,50 | 6,44 | 6,26 | 3,57 | 1,16 | 4,98 |
| | 4 | 1,51 | 4,74 | 4,03 | 0,65 | 0,07 | 0,15 | 1,85 |
| | 5 | 3,27 | 6,73 | 7,73 | 1,09 | 3,67 | 0,16 | 3,77 |
| | 6 | 2,96 | 6,27 | 6,51 | 0,67 | 3,07 | 3,90 | 3,89 |
| | 7 | 2,63 | 5,62 | 5,34 | 0,75 | 0,08 | 0,75 | 2,53 |
| | 8 | 1,69 | 5,82 | 4,25 | 4,92 | 0,24 | 2,65 | 3,26 |
| | 9 | 2,42 | 4,38 | 6,02 | 3,80 | 1,02 | 7,36 | 4,17 |
| | 10 | 6,57 | 6,23 | 2,33 | 1,65 | 1,57 | 6,84 | 4,20 |
| | 11 | 4,56 | 5,43 | 7,43 | 9,48 | 1,75 | 5,25 | 5,65 |
| Group of elderly practicing Nordic Walking (GHA) | 1 | 3,35 | 5,58 | 6,10 | 0,90 | 1,29 | 5,79 | 3,83 |
| | 2 | 4,88 | 5,68 | 5,44 | 4,15 | 8,32 | 2,12 | 5,10 |
| | 3 | 6,35 | 5,69 | 5,41 | 0,59 | 9,97 | 0,19 | 4,70 |
| | 4 | 2,48 | 5,38 | 6,06 | 6,56 | 0,18 | 5,11 | 4,29 |
| | 5 | 5,99 | 6,64 | 2,27 | 2,91 | 5,62 | 8,07 | 5,25 |
| | 6 | 2,48 | 4,18 | 3,97 | 4,45 | 1,75 | 8,26 | 4,18 |
| | 7 | 4,56 | 4,36 | 7,63 | 0,49 | 2,19 | 2,07 | 3,55 |
| | 8 | 2,94 | 5,51 | 7,81 | 2,14 | 2,27 | 1,92 | 3,76 |
| | 9 | 5,49 | 4,90 | 7,52 | 2,71 | 6,53 | 1,16 | 4,72 |
| | 10 | 4,94 | 6,47 | 6,31 | 4,06 | 0,99 | 4,38 | 4,53 |
| | 11 | 5,27 | 5,74 | 1,67 | 2,50 | 5,10 | 7,14 | 4,57 |
| | 12 | 3,68 | 4,85 | 6,28 | 2,04 | 6,67 | 2,31 | 4,31 |
| | 13 | 3,67 | 5,72 | 2,80 | 3,06 | 4,68 | 4,92 | 4,14 |
| | 14 | 5,51 | 6,01 | 1,38 | 6,34 | 9,03 | 8,79 | 6,18 |
| | 15 | 3,38 | 5,65 | 6,69 | 6,51 | 0,91 | 1,24 | 4,06 |
| | 16 | 5,84 | 4,66 | 6,99 | 1,89 | 9,64 | 1,18 | 5,03 |
| | 17 | 5,89 | 7,03 | 0,13 | 2,85 | 6,85 | 5,89 | 4,77 |
| | 18 | 4,74 | 5,11 | 2,48 | 6,46 | 7,09 | 8,78 | 5,78 |
| | 19 | 6,08 | 4,45 | 4,78 | 2,86 | 5,51 | 3,39 | 4,51 |
| | 20 | 4,84 | 6,33 | 3,84 | 6,98 | 3,01 | 8,01 | 5,50 |
